# Artificial intelligence assisted detection of superficial esophageal squamous cell carcinoma in white-light endoscopic images by using a generalized system

**DOI:** 10.1007/s12672-023-00694-3

**Published:** 2023-05-19

**Authors:** Yadong Feng, Yan Liang, Peng Li, Qigang Long, Jie Song, Mengjie Li, Xiaofen Wang, Cui-e Cheng, Kai Zhao, Jifeng Ma, Lingxiao Zhao

**Affiliations:** 1 Department of Gastroenterology, Zhongda Hospital Southeast University, 87 Dingjiaqiao Street, Nanjing, 210009 China; 2 Suzhou Institute of Biomedical Engineering and Technology, Chinese Academy of Sciences, 88 Keling Road, Suzhou, 215163 China; 3 Department of Gastroenterology, the Affiliated Changshu Hospital of Nantong University, Changshu No. 2 People’s Hospital, 18 Taishan Road, Suzhou, 215500 China; 4 Department of Gastroenterology, Changzhou Jintan First People’s Hospital Affiliated to Jiangsu University, 500 Jintan Avenue, Jintan, 210036 China; 5 School of Biomedical Engineering (Suzhou), Division of Life Sciences and Medicine, University of Science and Technology of China, 96 Jinzhai Road, Hefei, 230026 China; 6 Department of Gastroenterology, General Global Maanshan 17th Metallurgy Hospital, 828 West Hunan Road, Maanshan, 243011 China

## Abstract

**Background:**

The use of artificial intelligence (AI) assisted white light imaging (WLI) detection systems for superficial esophageal squamous cell carcinoma (SESCC) is limited by training with images from one specific endoscopy platform.

**Methods:**

In this study, we developed an AI system with a convolutional neural network (CNN) model using WLI images from Olympus and Fujifilm endoscopy platforms. The training dataset consisted of 5892 WLI images from 1283 patients, and the validation dataset included 4529 images from 1224 patients. We assessed the diagnostic performance of the AI system and compared it with that of endoscopists. We analyzed the system's ability to identify cancerous imaging characteristics and investigated the efficacy of the AI system as an assistant in diagnosis.

**Results:**

In the internal validation set, the AI system's per-image analysis had a sensitivity, specificity, accuracy, positive predictive value (PPV), and negative predictive value (NPV) of 96.64%, 95.35%, 91.75%, 90.91%, and 98.33%, respectively. In patient-based analysis, these values were 90.17%, 94.34%, 88.38%, 89.50%, and 94.72%, respectively. The diagnostic results in the external validation set were also favorable. The CNN model’s diagnostic performance in recognizing cancerous imaging characteristics was comparable to that of expert endoscopists and significantly higher than that of mid-level and junior endoscopists. This model was competent in localizing SESCC lesions. Manual diagnostic performances were significantly improved with the assistance by AI system, especially in terms of accuracy (75.12% vs. 84.95%, *p* = 0.008), specificity (63.29% vs. 76.59%,* p* = 0.017) and PPV (64.95% vs. 75.23%, *p* = 0.006).

**Conclusions:**

The results of this study demonstrate that the developed AI system is highly effective in automatically recognizing SESCC, displaying impressive diagnostic performance, and exhibiting strong generalizability. Furthermore, when used as an assistant in the diagnosis process, the system improved manual diagnostic performance.

**Supplementary Information:**

The online version contains supplementary material available at 10.1007/s12672-023-00694-3.

## Introduction

Esophageal cancer (EC) is the sixth most common cause of mortality of cancers worldwide [1]. Esophageal squamous cell carcinoma (ESCC) is the most predominant histological subtype of EC, mainly distributed in Eastern Asia [1–4]. Due to great differences in over 5 year survival [5–7], it is clinically significant to detect early-stage ESCCs. In most hospitals worldwide, white light imaging (WLI) is the most common endoscopic modality for detecting early-stage ESCC, which is always presented as a flat lesion and is defined as the superficial ESCC (SESCC) [5–7]. Due to lack of obvious morphological changes, WLI-based diagnosis for SESCC is an experience-dependent procedure, and there remain some misdiagnoses of SESCC by those unexperienced endoscopists.

For the aim to improve detection of SESCC, a strategy of artificial intelligence (AI) aided detection has been adopted. And, some researchers have studied AI-assisted diagnosis of ESCC and have made remarkable progress [8–19] (see Table S1 for brief introduction of these published models). However, such AI models used are usually trained using image data from one specific endoscopy system, and lack of universal properties. Since various endoscopy platforms are widely used, the role of currently available AI reading systems as an adjuvant method for the endoscopic detection of SESCC is limited.

For the aim of providing a working scheme with general compatibility, we developed and validated an AI model for automatically diagnosing SESCC by using WLI images from Olympus (Japan) and Fujifilm (Japan) endoscopy systems.

## Materials and methods

### Study design

**Fig. 1:**
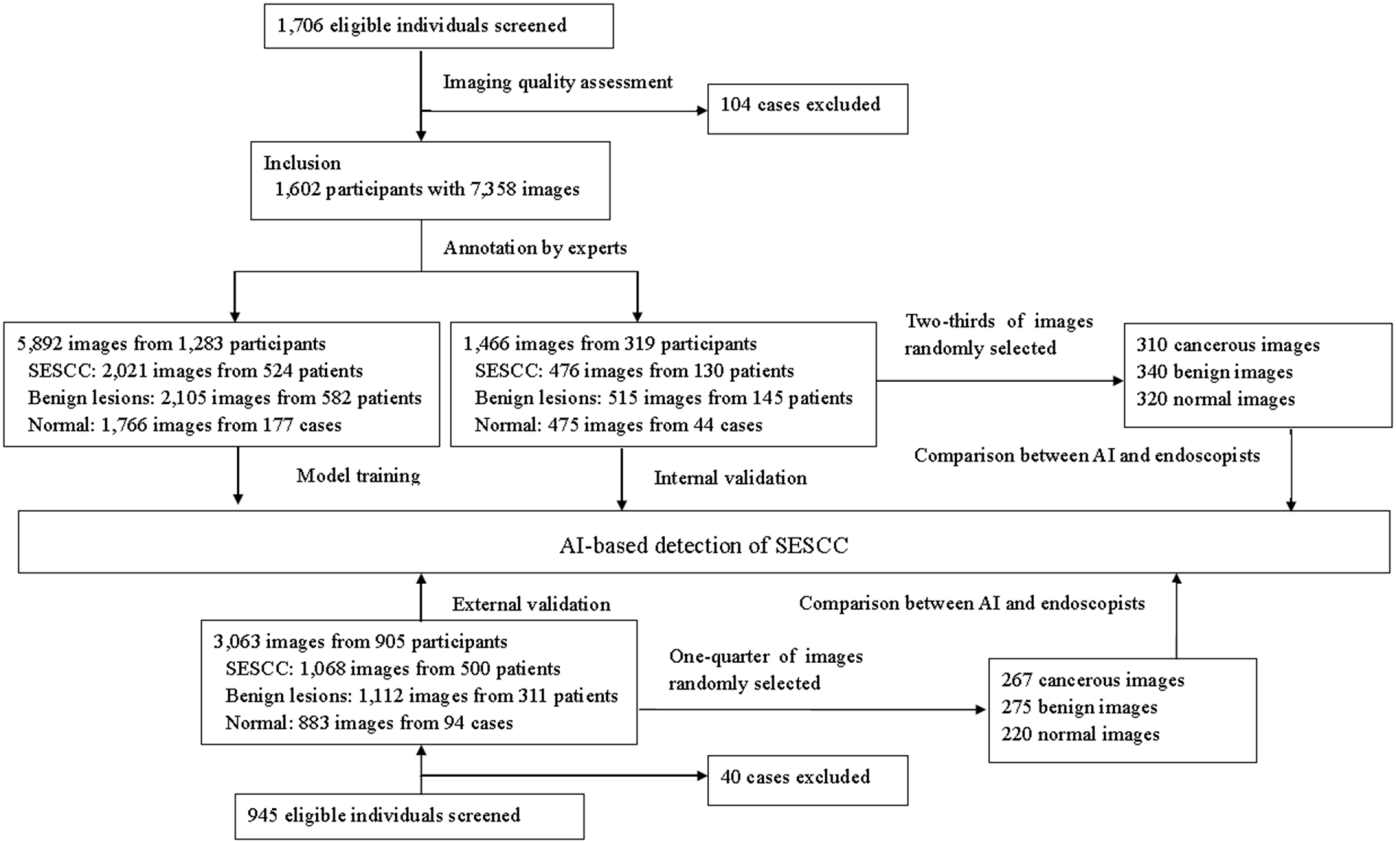
Flowchart of this study. *AI* artificial intelligence, *SESCC* superficial esophageal squamous cell carcinoma

Since January 2020, this study has been conducted in twelve hospitals in Jiangsu Province, China, including Zhongda Hospital Southeast University, Changzhou Jintan First People’s Hospital Affiliated to Jiangsu University, Changshu No.2 people’s Hospital Affiliated to Xuzhou Medical University, First Affiliated Hospital to Nanjing Medical University, Xuzhou First People’s Hospital, the Affiliated Hospital of Yangzhou University, Jiangning People’s Hospital Affiliated to Nanjing Medical University, Huai’an First People’s Hospital Affiliated to Nanjing Medical University, Taizhou People’s Hospital Affiliated to Nantong University, Jiangyin People’s Hospital Affiliated to Xuzhou Medical University, Lianshui County People’s Hospital and Jingjiang County People’s Hospital. This work was in cooperation with Suzhou Institute of Biomedical Engineering and Technology, Chinese Academy of Sciences, China. The flowchart of this study is shown in Fig. 1.

### Patient recruitment

Potential eligible participants were identified by searching patients with endoscopically and histopathologically confirmed SESCC, and WLI-imaging confirmed benign superficial esophageal lesions (esophageal leukoplakia, benign esophageal erosion, heterotopic gastric mucosa in esophagus, reflux esophagitis, acute esophageal mucosal injury) from medical databases of each participating endoscopy center. The histopathological diagnosis of ESCC was defined according to the criteria of the WHO 2019 [20].

Patients with high-quality WLI images and histopathologically confirmed SESCCwere finally recruited. Those patients with poor-quality images and/or indeterminacy of histopathological outcomes were excluded. Images from Zhongda Hospital Southeast University and First Hospital Affiliated to Nanjing Medical University were used as the training set and internal validation set, respectively. Images from other ten hospitals were used as the external validation set.

### Data collection, datasets and annotation

WLI endoscopic images were captured from Olympus (GIF­HQ290, GIF­H290, or GIF­Q260J, EVIS LUCERA ELITECV­290/CLV­290SL, CV-260, Japan) and Fujifilm (EG-L590ZW, EG-600ZW, VP­4450HD/XL­4450, VP-7000/BL7000, Japan) endoscopy systems. A total of 10,421 WLI endoscopic images of SESCC, benign lesions and normal esophageal mucosa from 2651 participants were initially obtained. After quality assessment by three expert endoscopists (who have endoscopic volumes of more than 3000 cases annually and at least 10 year experiences as endoscopist), 9686 WLI endoscopic images from 2507 participants were finally included. Data distribution is also shown in Fig. 1, including: (1) 5892 images from 1283 participants were used as the training set, (2) 1466 images from 319 participants were used as the internal validation set; (3) 3063 images from 905 participants were used as the external validation. Detailed information of data types and sources is listed in Table 1.

**Table1:** Information of data distribution of participants, images stratified by endoscopy platforms

	Training set		Internal validation set		External validation set	
	Olympus	Fujifilm	Olympus	Fujifilm	Olympus	Fujifilm
Participants, (n)						
SESCC	318	206	77	53	402	98
Benign lesions	341	241	89	56	181	130
Normal mucosa	131	46	34	10	61	33
Total	790	493	200	119	644	261
Image numbers, (n)						
SESCC	1245	776	308	168	868	200
Benign lesions	1304	801	339	176	702	410
Normal mucosa	1283	483	352	123	558	325
Total	3832	2060	999	467	2128	935

*SESCC* superficial esophageal squamous cell carcinoma

Lesions, including SESCC, benign lesions and normal esophageal mucosa, were labeled by five competent endoscopists (who have at least 5 year experiences as the endoscopist) independently. These annotations were then reviewed and confirmed by three expert endoscopists. The labeling confirmed by expert endoscopists was set as the gold standard for further study.

Morphological properties of lesions, including redness, nodule, white coat covering, location, macroscopic types and lesion size, were identified and recorded. Histopathological characteristics were also reviewed. Lesion locations and macroscopic types were determined according to existing diagnostic criteria [21, 22]. Endoscopic features, including macroscopic types, tumor sizes and luminal circumferential extension, of the enrolled SESCC are listed as Table 2.

**Table 2:** Endoscopic features of all enrolled SESCC cases

	Training set		Internal validation set		External validation set	
	Olympus (n = 318)	Fujifilm (n = 206)	Olympus (n = 77)	Fujifilm (n = 53)	Olympus (n = 402)	Fujifilm (n = 98)
Macroscopic type^a^, n						
Type 0-IIa	3	4	0	1	3	0
Type 0-IIb	260	118	65	37	322	79
Type 0-IIc	8	11	1	1	11	3
Mixed type	47	73	11	14	66	16
Type 0-IIa + IIc	5	13	0	2	3	4
Type 0-IIb + IIc	11	16	2	5	26	7
Type 0-IIb + IIa	19	20	6	3	26	3
Type 0-IIa + IIb	10	22	3	4	9	2
Type0-IIc + IIb	2	2	0	0	2	0
Tumor size, (cm) (average ± SD)	2.24 ± 1.32	2.54 ± 1.60	2.28 ± 1.43	2.47 ± 1.57	2.17 ± 1.34	2.18 ± 1.53
≥ 2.0 cm, n	104	93	26	24	132	43
< 2.0 cm, n	214	113	51	29	270	55
Circumferential extension, n						
≤ 1/4	179	79	50	22	244	53
1/4–1/2	75	54	10	12	96	33
1/2–3/4	51	46	11	13	49	9
> 3/4	13	27	6	6	13	3

*SESCC* superficial esophageal squamous cell carcinoma

^a^Macroscopic types were defined according to the Paris classification, type 0-IIa: slightly elevated type, type 0-IIb: flat type, type 0-IIc: slightly depressed type; SD: standard deviation

### Construction and validation of our AI system

The classification performances of widely-utilized networks, namely VGG16, InceptionV3, ResNet50, ResNeXt50, and DenseNet121, on white-light endoscopic images of the esophagus were assessed. And, the ResNet50 network exhibited the highest value of the area under the receiver operating characteristic (ROC) curve with superior classification performance and considerably higher sensitivity than the other models (see Table S2). Accordingly, the ResNet50 network is chosen as the fundamental framework for further model construction.

A novel Bilinear Pooling Attention Network (named BPAN) was proposed, and we developed a two-step diagnosis procedure based on this deep convolutional neural network (CNN) for the identification and localization of SESCC lesions under WLI. The architecture of BPAN, which is a classification network that categorizes images into three classes, is shown in Supplementary Figure S1. Details of the BPAN model design are elaborated in Supplementary content 1 and Supplementary Figure S2, which indicates that a two-category network that uses heat maps to display lesions in images classified as cancer and non-cancer. The primary aim was to identify SESCC lesions in WLI images, and the secondary aim was to delineate possible lesion margins. The diagnosis of SESCC, benign lesions and normal esophageal mucosa was determined by the predictive probability scores. The image was diagnosed as cancerous when the probability value surpassed the threshold value of 0.5. If any image of a patient was diagnosed as cancerous, the patient would be regarded as a cancer case. Only when all images of a patient were diagnosed as noncancerous, the patient was judged as a noncancer case.

The average inference time was 46 ms for per-image recognition and classification, and was 152 ms for producing a heat map in a single image. An independent internal validation set and an external validation set were used to evaluate the performance of the trained BPAN model.

### Comparison of diagnostic performances between our AI system and endoscopists

Two-thirds and one-quarter of images were randomly selected from the internal and external validation set, respectively, and were mixed and de-identified. The diagnostic capability of the AI-assisted diagnosis system was compared to those of endoscopists by using the internal and external validation data sets. Three senior (with clinical experience of more than 10 years as endoscopist), two middle-level (with clinical experience of 5–10 years as endoscopist) and three junior (with clinical experience of less than 5 years as endoscopist) endoscopists, who were not involved in the imaging labeling and masked to the endoscopic and histopathological results of esophageal lesions were invited to participate in further assessment. In the first stage, they made their independent diagnosis of whether there were SESCC lesions without AI assistance. Four weeks later, the same task was conducted again in an AI-assisted manner to evaluate whether the AI reading could improve endoscopists’ diagnostic performances. In this stage, the endoscopists were allowed to access the results given by the automatic reading of our AI system. The diagnostic measures of the endoscopists were compared between two diagnosis modalities. In addition, the diagnostic performances in identifying cancer-related characteristics were also evaluated and compared.

### Outcome measures

The diagnostic performances of the AI system and endoscopists (senior, mid-level and junior) for identifying SESCC were evaluated in terms of sensitivity, specificity, accuracy, positive predictive value (PPV) and negative predictive value (NPV) with the 95% confidence interval (CI) values. The Dice coefficient was calculated to evaluate the performance of our AI system for the localization of SESCC. The ROC curve and the area under ROC curve (AUC) were used to access the comprehensive diagnostic performance of our AI system.

### Statistical analyses

The two-sided McNemar test was used to compare diagnostic measures between the endoscopists of different levels and our AI system. Interobserver agreement of the endoscopists and our AI system for diagnosis of SESCC was assessed by the Cohen kappa coefficient. Additionally, the differences of endoscopists with or without the AI assistance were compared using the two-sided Wilcoxon signed-rank test and paired t test wherever applicable. A *p* value < 0.05 was considered to be statistically significant. Statistical analyses were performed using the SPSS software version 22.0.

## Results

### Diagnostic performances in identifying SESCC

**Fig. 2:**
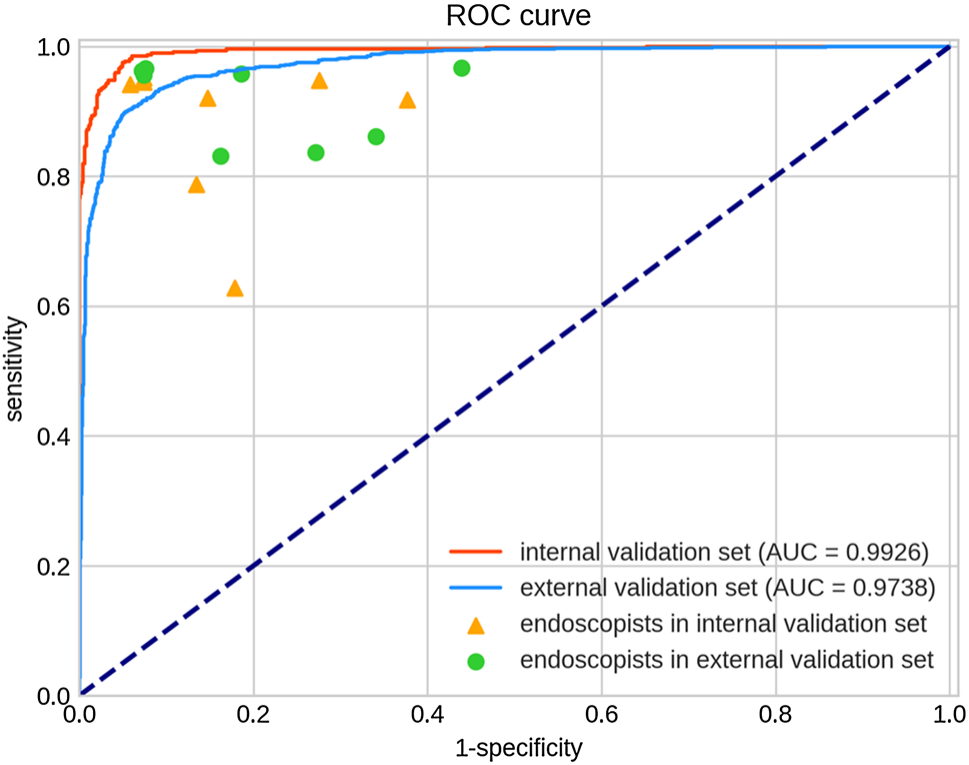
Receiver operating characteristic (ROC) curves of the AI model and results of endoscopists in the internal and external image validation sets. ROC curves were performed by using sensitivity against (1-specificity). The area under ROC curve (AUC) values were 0.993 and 0.974, respectively, in internal and external validation set. The diagnostic performance of the AI model was higher than that of all endoscopists in internal validation set, and superior to that of most endoscopists in external validation set. *AI* artificial intelligence

Our AI system demonstrated promising diagnostic performances of per-image analysis in internal (AUC = 0.993) and external (AUC = 0.974) validation sets (Fig. 2). The accuracy, sensitivity, specificity, PPV and NPV were 91.75% (95% CI 90.34–93.16%), 96.4% (95% CI 94.48–98.00%), 95.35% (95% CI 93.80–96.54), 90.91% (95% CI 87.98–93.20) and 98.33% (95% CI 97.25–99.01), respectively, in the internal validation set. And, they were 88.38% (95% CI 87.25–89.51%), 90.17% (95% CI 88.19–91.86%), 94.34% (95% CI 93.21–95.29%), 89.50% (95% CI 87.47–91.23%) and 94.72% (95% CI 93.62–95.64%), respectively, in the external validation set.

Then, per-patient analysis was performed. In the internal validation set, 129 out of 130 patients of SESCC and 160 out of 189 patients of normal/benign lesions were correctly detected. The sensitivity, specificity and accuracy were 99.23% (95% CI 95.16–99.96%), 84.66% (95% CI 78.54–89.32%) and 90.60% (95% CI 87.39–93.80%), respectively. In the external validation set, 458 out of 500 patients of SESCC and 360 out of 405 patients of normal/benign lesions were correctly recognized as cancerous and noncancerous cases, respectively. Sensitivity, specificity and accuracy were 91.60% (95% CI 88.73–93.81%), 88.89% (95% CI 85.32–91.70%) and 88.38% (95% CI 87.24–98.51%), respectively, in this cohort. These results are outlined in Table 3.

**Table 3:** Diagnostic performance of the AI model in identifying SESCC

	Internal validation set		External validation set	
	Per-patient	Per-image	Per-patient	Per-image
Accuracy (95% CI)	90.60% (87.39–93.80)	91.75% (90.34–93.16)	90.39% (88.47–92.31)	88.38% (87.24–89.51)
Sensitivity (95% CI)	99.23% (95.16–99.96)	96.64% (94.48–98.00)	91.60% (88.73–93.81)	90.17% (88.19–91.86)
Specificity (95% CI)	84.66% (78.54–89.32)	95.35% (93.80–96.54)	88.89% (85.32–91.70)	94.34% (93.21–95.29)
PPV (95% CI)	81.65% (74.54–87.18)	90.91% (87.98–93.20)	91.05% (88.13–93.33)	89.50% (87.47–91.23)
NPV (95% CI)	99.38% (96.07–99.97)	98.33% (97.25–99.01)	89.55% (86.04–92.29)	94.72% (93.62–95.64)

*AI* artificial intelligence, *SESCC* superficial esophageal squamous cell carcinoma, *PPV* positive predictive value, *NPV* negative predictive value

### Comparison of diagnostic performances between our AI system and endoscopists

In the internal validation set, the diagnostic performance of the AI-assisted detection was comparable to those of expert endoscopists and significantly higher than those of the non-expert groups. In the external validation set, our AI system achieved the highest specificity and PPV. The accuracy was similar to that of the senior group. The sensitivity was lower than that of senior endoscopists, but was similar to those of mid-level endoscopists and junior endoscopists. The diagnostic outcomes of the senior group were higher than those of the other two groups. Similarly, there were also significant differences between mid-level and junior level endoscopists. These results are shown in Table 4 and S3.

**Table 4:** Diagnostic performance of the AI model and endoscopists in identifying of SESCC

	Internal validation set				External validation set			
	AI	Senior	Mid-level	Junior	AI	Senior	Mid-level	Junior
Accuracy (%)	90.60	87.15 (1.33)	75.08 (2.44)	63.11 (4.46)	90.39	89.17 (0.11)	77.13 (2.34)	67.04 (1.28)
Sensitivity (%)	99.23	96.41 (0.44)	89.62 (5.99)	90.00 (9.32)	91.60	96.80 (0.53)	92.80 (7.35)	93.07 (4.62)
Specificity (%)	84.66	80.78 (2.14)	65.08 (0.00)	44.62 (12.43)	88.89	79.75 (0.89)	57.78 (3.84)	34.90 (7.46)
PPV (%)	81.65	77.56 (1.89)	63.80 (1.54)	53.11 (3.50)	91.05	85.25 (0.48)	73.08 (0.23)	63.90 (1.60)
NPV (%)	99.38	97.04 (0.31)	90.26 (5.14)	88.12 (7.13)	89.55	95.29 (0.69)	87.76 (11.25)	82.13 (9.60)

Outcomes from manual diagnoses are expressed as mean ± standard deviation, which is listed in the parenthesis

*AI* artificial intelligence, *SESCC* superficial esophageal squamous cell carcinoma, *PPV* positive predictive value, *NPV* negative predictive value

The interobserver agreement of diagnoses between our AI system and endoscopists was assessed and listed in Table 5. Perfect (kappa score, 0.918–0.967), moderate (kappa score, 0.540–0.579) and fair agreements (kappa score, 0.305–0.505) were present in senior, mid-level and junior endoscopists, respectively. Additionally, interobserver agreement between our AI system and expert endoscopists (kappa score, 0.708–0.786) was higher than that of the AI system and mid-level endoscopists (kappa score, 0.564–0.711), and further superior to that of our AI system and junior endoscopists (kappa score, 0.355–0.600).

**Table 5:** Interobserver agreements between endoscopists and the AI model

	AI	S1	S2	S3	M1	M2	J1	J2	J3
AI	–	0.747^a^	0.781^a^	0.786^a^	0.711^a^	0.566^a^	0.447^a^	0.461^a^	0.600^a^
S1	0.708^b^	–	0.930^a^	0.918^a^	–	–	–	–	–
S2	0.731^b^	0.967^b^	–	0.948^a^	–	–	–	–	–
S3	0.720^b^	0.965^b^	0.958^b^	–	–	–	–	–	–
M1	0.658^b^	–	–	–	–	0.540^a^	–	–	–
M2	0.564^b^	–	–	–	0.579^b^	–	–	–	–
J1	0.355^b^	–	–	–	–	–	–	0.305^a^	0.505^a^
J2	0.455^b^	–	–	–	–	–	0.356^b^	–	0.448^a^
J3	0.420^b^	–	–	–	–	–	0.408^b^	0.393^b^	–

S1,2,3, senior endoscopist 1,2,3; M1,2, mid-level endoscopist 1,2; J1,2,3, junior endoscopist1, 2, 3

*AI* artificial intelligence

^a^Internal validation set

^b^External validation set

Abilities in identifying cancer-related imaging characteristics by our AI system and manual reading were compared by stratifying patients with imaging-based morphological features and tumor locations. Our AI system showed a favorable performance for identifying different cancer conditions. Manual diagnoses showed higher sensitivities for detecting large lesions (> 2 cm) and lesions with obvious morphological changes. AI-assisted detection was significantly superior to mid-level and junior groups by presenting higher sensitivities in detecting small lesions and lesions without obvious morphological changes (see Table S4 and S5).

### Improvement of diagnostic performance by using AI assistance

**Fig. 3:**
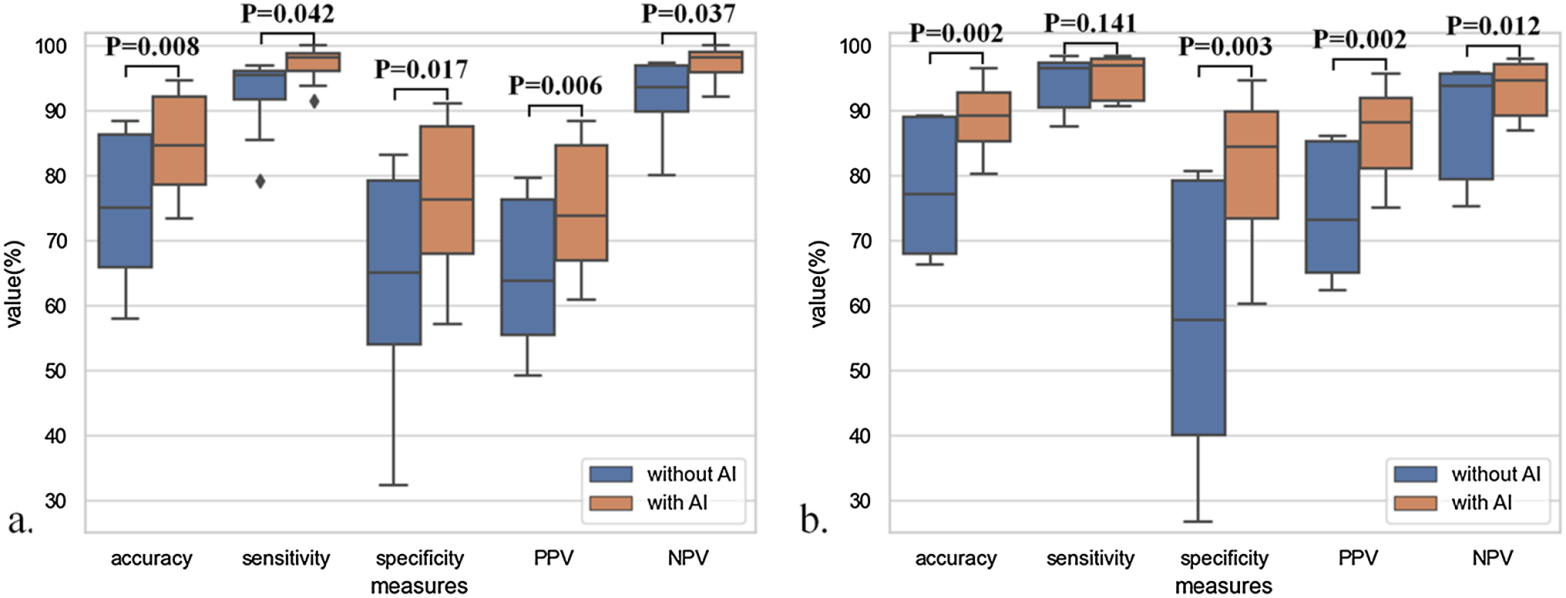
Comparison of manual diagnostic outcomes of endoscopists with or without AI reading as assistance. **a** Diagnostic outcomes from internal validation, accuracy, sensitivity, specificity, positive predictive value (PPV) and negative predictive value (PPV) were significantly increased by using AI model as assistant diagnosis; **b** Diagnostic measures from external validation, sensitivity was not statistically differed, accuracy, specificity, PPV and NPV were significantly improved. *AI* artificial intelligence

Diagnostic performances were compared between the pure manual diagnosis and that under the AI assistance, and the comparison results are shown in Fig. 3. In the internal validation set, all of the diagnostic measures of endoscopists were improved by presenting increases of evaluated diagnostic accuracy (75.12% vs. 84.95%, *p* = 0.008), sensitivity (92.31% vs. 97.12%, *p* = 0.042), specificity (63.29% vs. 76.59%, *p* = 0.017), PPV (64.95% vs. 75.23%, *p* = 0.006) and NPV (92.00% vs. 97.25%, *p* = 0.037). For the external validation set, the sensitivity (94.40% vs. 95.23%, *p* = 0.141) was not statistically differed. Other measures, including accuracy (77.86% vs. 89.03%, *p* = 0.002), specificity (57.44% vs. 81.39%, *p* = 0.003), PPV (74.30% vs. 86.85%, *p* = 0.002) and NPV (88.47% vs. 93.29%, *p* = 0.012), were significantly increased.

Detailed results for improving diagnostic performances of endoscopists are shown in Table 6. Setting the automatic reading by our AI system as the reference, diagnostic performances of mid-level and junior endoscopists were substantially elevated. For the senior group, the performance was also improved. In particular, the specificity of the junior group had the most significant improvement in the internal and external validation sets, which were 25.22% and 39.34%, respectively.

**Table 6:** Diagnostic performances of endoscopists in identifying of SESCC before/after referring to the results of AI model

	Accuracy %	Sensitivity %	Specificity %	PPV %	NPV %
Internal validation set					
Senior					
Before	87.15	96.41	80.78	77.56	97.04
After	93.42	99.49	89.24	86.45	99.60
Mid-level					
Before	75.08	89.62	65.08	63.80	90.26
After	79.15	95.77	67.73	67.12	95.93
Junior					
Before	63.11	90.00	44.62	53.11	88.12
After	80.36	95.64	69.84	69.41	95.79
All					
Before	75.12	92.31	63.29	64.95	92.00
After	84.95	97.12	76.59	75.23	97.25
External validation set					
Senior					
Before	89.17	96.80	79.75	85.25	95.29
After	94.80	98.13	90.70	92.99	97.50
Mid-level					
Before	77.13	92.80	57.78	73.08	87.76
After	86.97	94.10	78.15	84.45	91.99
Junior					
Before	67.04	93.07	34.90	63.90	82.13
After	84.64	93.07	74.24	82.32	89.93
All					
Before	77.86	94.40	57.44	74.30	88.47
After	89.03	95.23	81.39	86.85	93.29

*AI* artificial intelligence, *SESCC* superficial esophageal squamous cell carcinoma, *PPV* positive predictive value, *NPV* negative predictive value

Furthermore, as shown in Table 7, the interobserver agreements of endoscopists in mid-level and junior groups were also improved with the AI-assisted diagnosis. In the mid-level group, the individual kappa scores in internal and external validation sets were increased to 0.713 and 0.755, respectively. And the kappa values of three observers from junior group ranged from 0.708 to 0.807.

**Table 7:** Interobserver agreements of endoscopists after referring to the results of AI model

	S1	S2	S3	M1	M2	J1	J2	J3
S1	–	0.878^a^	0.872^a^	–	–	–	–	–
S2	0.873^b^	–	0.990^a^	–	–	–	–	–
S3	0.870^b^	0.980^b^	-	–	–	–	–	–
M1	–	–	–	–	0.713^a^	–	–	–
M2	–	–	–	0.755^b^	–	–	–	–
J1	–	–	–	–	–	–	0.807^a^	0.742^a^
J2	–	–	–	–	–	0.808^b^	–	0.762^a^
J3	–	–	–	–	–	0.708^b^	0.729^b^	–

S1,2,3, senior endoscopist 1,2,3; M1,2, mid-level endoscopist 1,2; J1,2,3, junior endoscopist 1,2,3

*AI* artificial intelligence

^a^Internal validation set

^b^External validation set

### Localization of SESCC regions

**Fig. 4:**
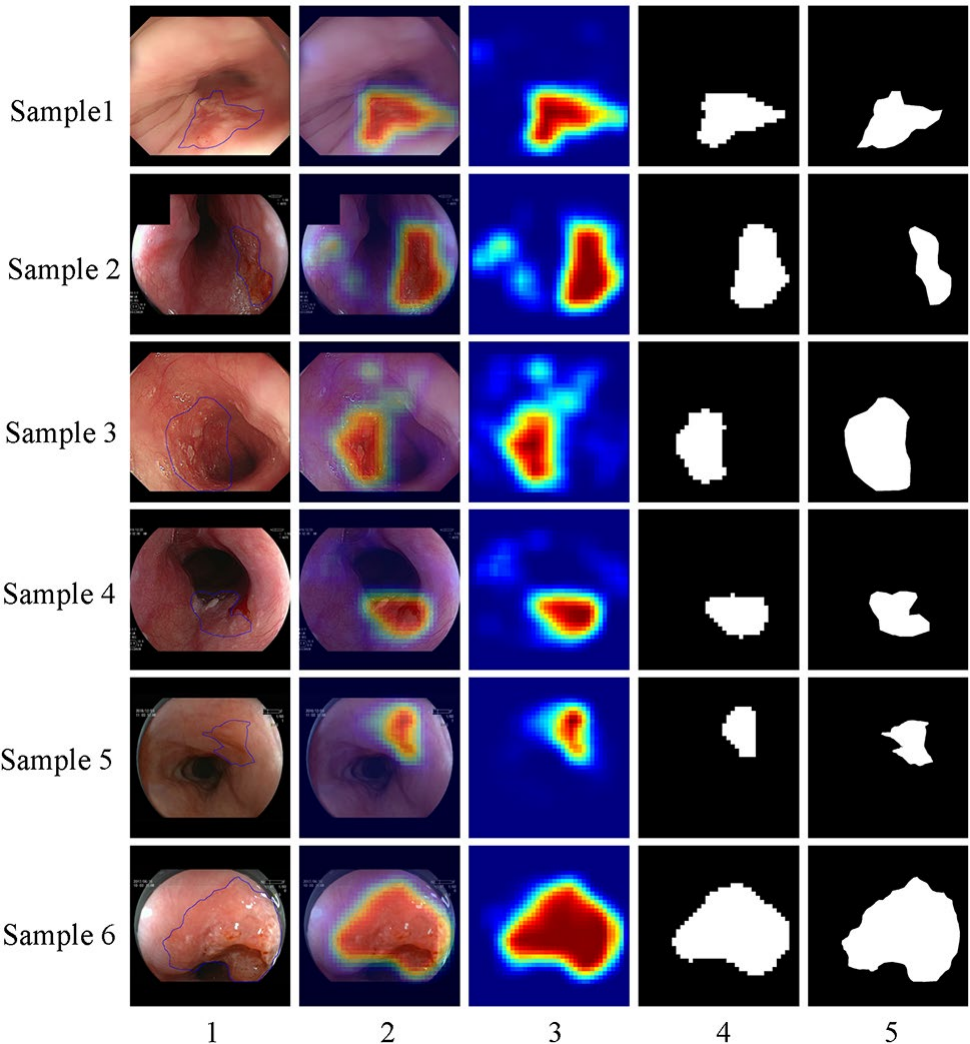
Results of AI-based localization of SESCC under WLI from internal and external validation. Sample 1–3: internal validation; Sample 4–6: external validation. Column 1: the original WLI images, where the lesion was manually marked by experienced endoscopists with blue delineation; Column 2: the locating result of lesion mapped by the probability heat map; Column 3: the pseudo-colored patch-based probability heat map generated by AI system; Column 4: the tumor region obtained by the cutoff value of 0.5; Column 5: the ground truth of lesion marked by experienced endoscopists. *AI* artificial intelligence, *SESCC* superficial esophageal squamous cell carcinoma, *WLI* white light imaging

Our BPAN model was designed to provide the heat map related to each input endoscopic image, which indicates probabilities of cancerous regions in the full image. Also, the ability of localizing possible SESCC lesions was assessed according to the gold standard manually created by expert endoscopists (see Supplementary content 2). Samples of AI-based localization of SESCC lesions in the internal and external validation data sets are shown in Fig. 4, respectively. These results suggested that our AI system using the proposed BPAN model had a favorable performance in identifying lesion regions of SESCC.

## Discussion

SESCC is prone to be misdiagnosed under WLI even by using a high-definition endoscopy. This is due to the fact that such a lesion usually lacks apparent morphological features [7]. In this study, we developed an AI system based on a novel CNN model with an excellent diagnostic performance for detecting SESCC in WLI endoscopic images. This CNN model has a strong diagnostic ability comparable to that of expert endoscopists, and is significantly higher than those of mid-level and junior endoscopists. To the best of our knowledgement, we firstly reported a generalized AI model, which is compatible with multiple endoscopy platforms, for detecting SESCC by using WLI images only.

Recently, several studies reported their researches in the WLI-based endoscopic detection of SESCC [8, 10, 11, 13, 16, 23, 24]. In these studies, endoscopic images in their datasets are captured using the Olympus endoscopy system only. The AI models used in the studies by Cai [11] and Horie [13] failed to perform detailed lesion localizations. Since imaging characteristics may vary significantly among different endoscopy systems, it is very difficult to guarantee the capability of a single AI model to handle image characteristics greatly variable. As a result, none of these AI models are clinically sufficient as the assistant diagnosis for multiple endoscopy systems. For this reason, we adopted a newly designed training strategy by using WLI images from Olympus and Fujifilm endoscopy system.

Taking the AI reading as the assistant diagnosis into account, an AI model with a high diagnostic accuracy for identifying lesions of interest is appreciated. In most previous studies [14, 23, 24], WLI images of normal esophageal mucosa were used as control data in model training, but benign or inflammatory lesions were neglected. Diagnostic accuracies from these studies ranged from 81 to 86.4%. Recently, Tang et al. [16] used a training cohort containing WLI images of normal esophageal mucosa and benign esophageal lesions. They achieved a higher diagnostic accuracy of 91.3% in their internal validation set. In this study, we adopted a similar strategy by collecting diverse benign images for training. Except for normal esophageal mucosa and reflux esophagitis, WLI images of other reddish, superficial benign esophageal mucosal lesions were also included for model training. The diagnostic performances, including diagnostic accuracy, sensitivity, specificity, PPV and NPV, of this AI system were some higher than those of other existing models [14, 23, 24]. For example, our model showed a diagnostic accuracy of 91.75% in differentiating SESCC from other lesions in the internal validation dataset. Our AI model is also superior to Lugol iodine chromoemdoscopy, which is most commonly used for detecting early-stage ESCC in clinical practice [25], by demonstrating a comparable sensitivity (96.64% vs. > 95%) and a significantly increased specificity (95.35% vs. 65%).

Abilities for recognizing detailed SESCC imaging characteristics were assessed for revealing possible mechanisms of diagnostic behaviors. The outcomes were higher than those of expert endoscopists and significantly superior to those of mid-level and junior endoscopists. This contributed to improve the diagnostic performance of the AI-based detection for SESCC. Also, our AI model was competent in localizing SESCC lesions. The most abnormal lesion site could be determined according to the area that contains highest probabilities in the probability heat map. Recently, Liu et al. [8] reported their study of AI-based delineation of SESCC margins under WLI. To the best of our knowledgement, Lugol iodine staining chromoendoscopy shows a higher accuracy in delineating SESCC margins than those of other endoscopic diagnostic techniques, such as WLI endoscopy and virtual chromoendoscopy. Accordingly, we aimed to profile regions of interest rather than the accurate delineation of lesion margin under WLI.

The most interest of this study is to determine whether the diagnostic efficiency would be improved by using the AI reading as the reference. Consistent with previous studies [13, 16], our study evaluated diagnostic performances of manual diagnosis by endoscopists while referring to results of the AI reading. Our study showed that the highest improvement was present in junior endoscopists with low endoscopy volumes. This cooperative diagnosis also led to an increase in the interobserver agreement among endoscopists with different diagnostic levels [14, 16]. Therefore, our AI-assisted approach is qualified as an auxiliary method for the WLI-based detection of SESCC by bridging the gaps of diagnostic performances among endoscopists with different levels. According to our results, the diagnostic performances of junior and mid-level endoscopists were some lower than that of AI system. This is due to that WLI-based diagnosis of SESCC is experience demanding, and they still need more real-world cases for training.

There are some limitations of this study. First, we used high-quality and still endoscopic images for model training, validation and assessment of the diagnostic efficacy. This is because clinical diagnosis can be achieved by using still images. And, typical still images are commonly used as training materials for novices. Second, because all images in the training set were labeled retrospectively, there may be some bias of data selection and inclusion. However, results of validation test from prospective enrolled data demonstrated that there was no significant limitation. Also, our AI model showed promising diagnostic performances in the multicenter image dataset. Third, endoscopic assessment of invasion depth is not set as one goal of this study. This is because such diagnosis is routinely performed by using optical enhanced magnifying endoscopy systems. Also, results from the currently available report yielded a rough outcome [24]. For this purpose, we have designed a prospective trial by using multiple imaging modalities.

## Conclusions

We developed a novel AI-assisted SESCC diagnosis system with a novel compatibility. This system showed an excellent diagnostic performance for the WLI-based detection of SESCC. Our AI model can be used as an assistant method for endoscopists to detect SESCC during daily endoscopic examinations. Meanwhile, further prospective validations are still needed to evaluate its role in the real clinical circumstance.

## Supplementary Information

Additional file 1.

Additional file 2.

Additional file 3.

Additional file 4.

Additional file 5.

Additional file 6.

Additional file 7.

Additional file 8.

Additional file 9.

## Data Availability

The datasets used during the current study are included in this article, and are available from the corresponding author on reasonable request.

## Code availability

The code is not publicly available at this time as the code is also part of our ongoing research.
