# Supplementary material for: Artificial intelligence assisted detection of superficial esophageal squamous cell carcinoma in white-light endoscopic images by using a generalized system
Source: Discov Oncol. 2023 May 19;14:73. doi: 10.1007/s12672-023-00694-3 (PMC10199153; doi:10.1007/s12672-023-00694-3)
Supplement: Supplementary file 1 — Additional file 1. [file 12672_2023_694_MOESM1_ESM.docx]

Supplementary content1.Construction of the AI system

Since SESCC lesions lack discriminative clinical characteristics under the WLI endoscopy, there are some difficulties for a conventional CNN model to identify SESCC with the WLI images. We firstly designed a global channel attention module based on the SENet. The CNN model could effectively extract features of the most discriminative lesion regions. In addition, the hierarchical bilinear pooling was added to the back of CNN model, which simultaneously enabled the inter-layer interaction of high-dimensional features and combination of multiple cross-layer bilinear features to improve the feature representation capability of model. The subtle imaging discrepancy between cancer and noncancer could be better differentiated by the proposed CNN model. The performance of CNN model was significantly improved after combining the bilinear pooling and attention mechanism.

A patch-based classification strategy was utilized for the localization of suspicious lesions in cancer images. In the training stage, the small image patch of size 224×224 pixels was randomly selected from the full image. Subsequently, image enhancement techniques, including rescaling, rotation, translation, flip, as well as contrast, brightness hue and saturation jittering, were randomly adopted to improve the generalization ability of model. The category of each image patch was determined by the delineations of expert endoscopists. An image patch was labeled as positive sample only if more than 80% of its pixels were included in the delineation. Additionally, when less than 20% pixels of the image patch were included in the delineation, it was labeled as negative sample. Other image patches were excluded.

During the training process, a batch size of 64 was employed. The model was optimized using the FocalLoss loss function, while the weight updates were performed using the SGD optimizer with a default momentum of 0.9. Moreover, in order to improve the feature extraction process, we utilized pre-trained weight parameters and set the learning rate to le-4. The training process implemented an early stopping mechanism. The PyTorch framework was used for the model programming, whereas the experiment is conducted on an Nvidia Tesla V100 GPU server with a 32G memory.

During the evaluation, the full image was sampled equidistantly into a set of overlapping patches of size 224×224 with an offset of 20 pixels. The cancer probability of each image patch was then estimated by the trained CNN model. Subsequently, the probability heat map of cancer image was compiled by using the cancer probability value. For the tumor margin, a threshold t regarding cancer probability was taken into account. Only if the pixel-based cancer probability exceeded t, the corresponding region would be regarded as tumor in cancer image. The tumor margin was obtained by using the cutoff value of 0.5 in this study. Finally, the location map of lesion was derived based on the high cancer probability value more than t.
