# Supplementary material for: Artificial intelligence assisted detection of superficial esophageal squamous cell carcinoma in white-light endoscopic images by using a generalized system
Source: Discov Oncol. 2023 May 19;14:73. doi: 10.1007/s12672-023-00694-3 (PMC10199153; doi:10.1007/s12672-023-00694-3)
Supplement: Supplementary file 2 — Additional file 2. [file 12672_2023_694_MOESM2_ESM.docx]

Supplementary content 2.AI-based localization and delineation of SESCC regions

When any image of SESCC was detected by AI model, the suspicious SESCC region was highlighted in red color in the output heat map. The redder color indicated the higher cancer probability. The boundary of the suspicious lesion was determined by the cancer probability value more than 0.5. The Dice coefficient was calculated according to the lesion boundary delineation by experienced endoscopists as reference. And, the mean Dice coefficients were 0.74 and 0.72, respectively, in the internal and external validation sets.
