# Supplementary figures and images for: Artificial intelligence assisted detection of superficial esophageal squamous cell carcinoma in white-light endoscopic images by using a generalized system
Source: Discov Oncol. 2023 May 19;14:73. doi: 10.1007/s12672-023-00694-3 (PMC10199153; doi:10.1007/s12672-023-00694-3)

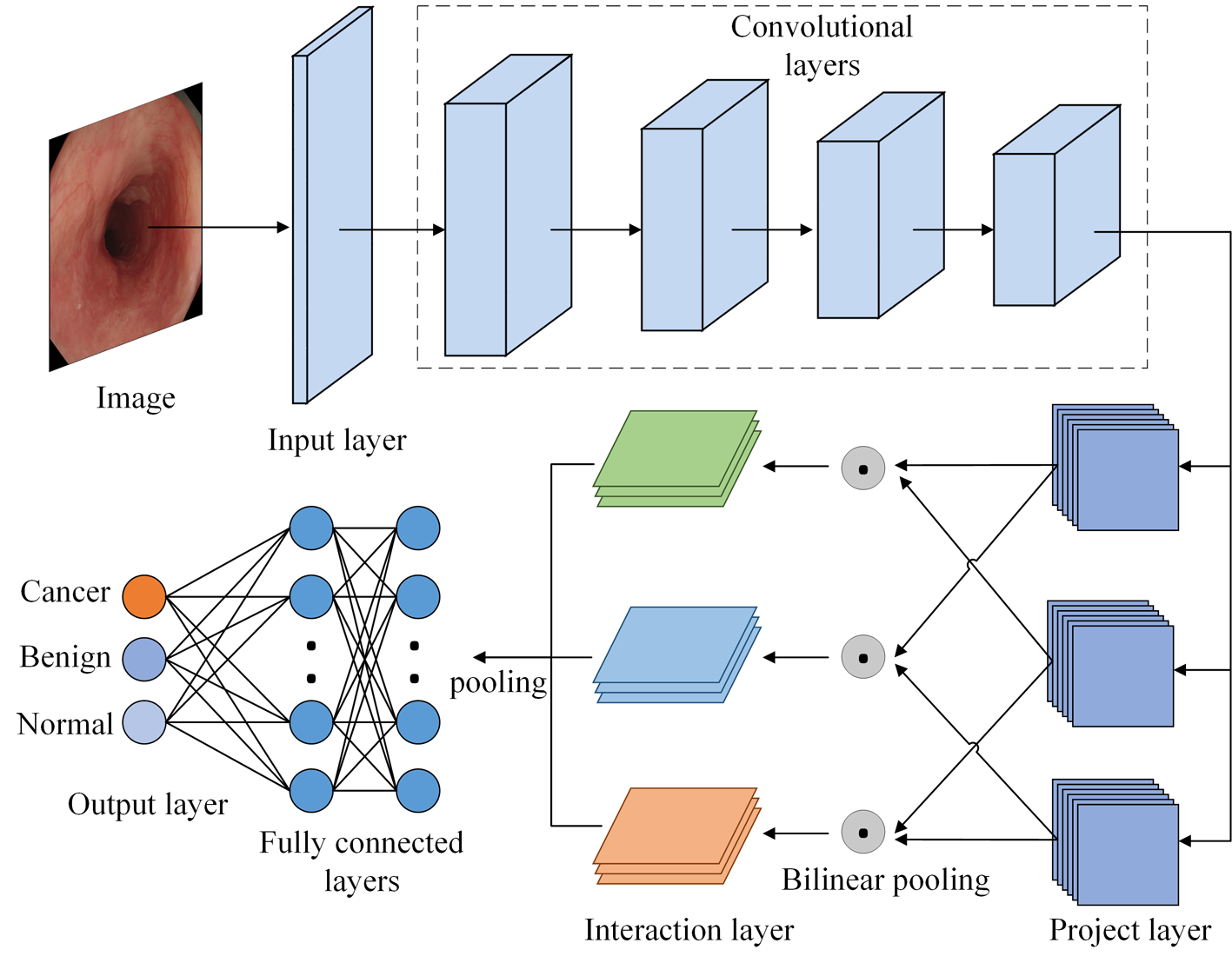

Supplement: Supplementary file 3 — Additional file 3. [file 12672_2023_694_MOESM3_ESM.tif]

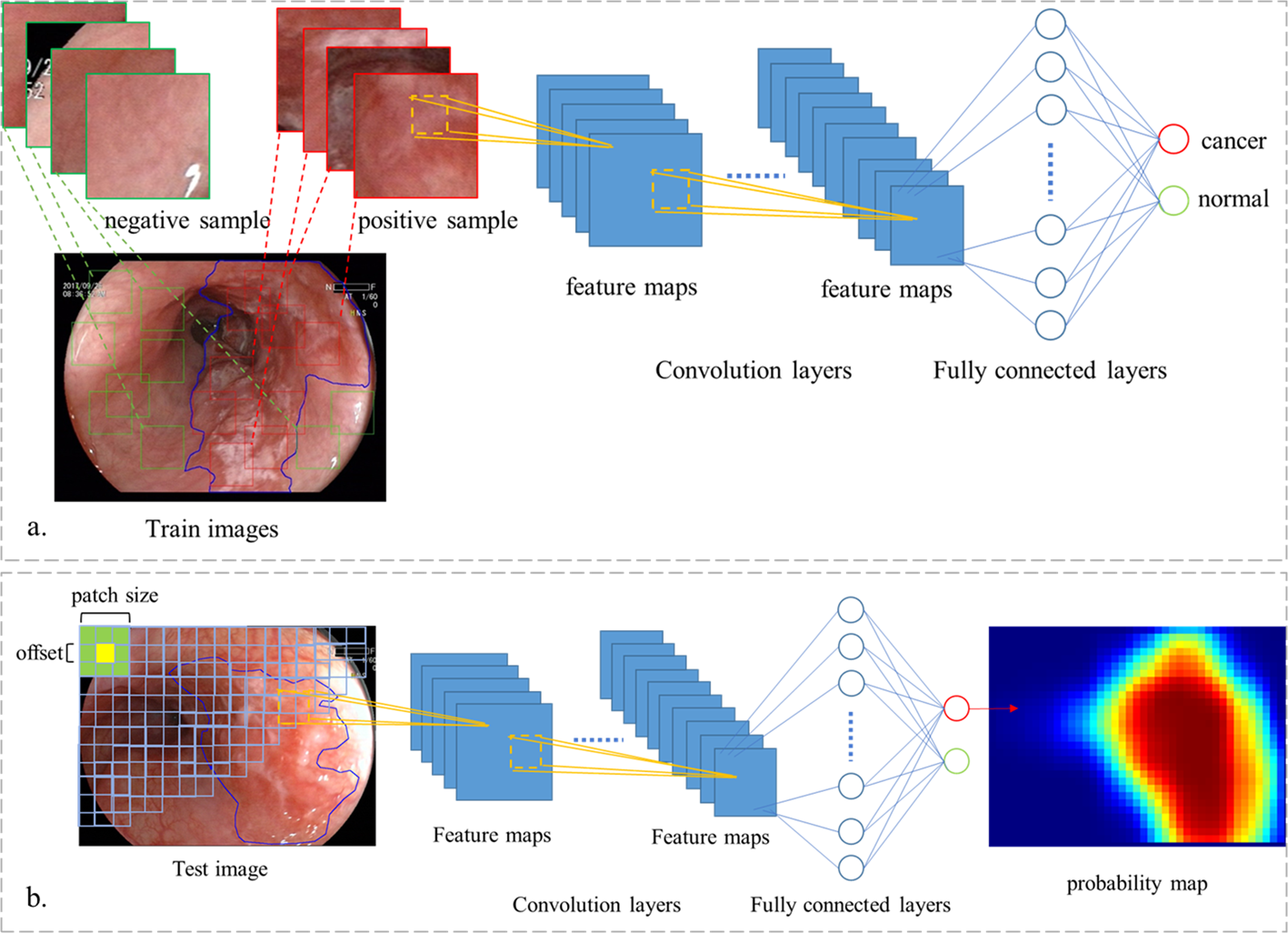

Supplement: Supplementary file 4 — Additional file 4. [file 12672_2023_694_MOESM4_ESM.tif]
