# Supplementary material for: Artificial intelligence assisted detection of superficial esophageal squamous cell carcinoma in white-light endoscopic images by using a generalized system
Source: Discov Oncol. 2023 May 19;14:73. doi: 10.1007/s12672-023-00694-3 (PMC10199153; doi:10.1007/s12672-023-00694-3)
Supplement: Supplementary file 5 — Additional file 5. [file 12672_2023_694_MOESM5_ESM.docx]

Table S1.Introduction of published literatures relevant to AI-assisted endoscopic diagnosis of esophageal squamous lesions

| Authors | Year | Endoscope platform | Modalities | Disease | Performance | | | | |
| --- | --- | --- | --- | --- | --- | --- | --- | --- | --- |
|  |  |  |  |  | Sensitivity | Specificity | PPV | NPV | Accuracy |
| Liu W, et al ^[8]^ | 2022 | Olympus | WLI | early ESCC | 92.6% | 80.0% | 81.8% | 91.3% | 85.7% |
| Yuan XL, et al ^[9]^ | 2022 | Olympus | WLI  non-ME NBI  iodine staining  ME NBI | SESCC | 96.9% | 83.1% | NA | NA | 91.1% |
| Yang XX, et al ^[10]^ | 2021 | Pentax | non-ME  ME-OE | early ESCC  advanced esophageal cancer | 97.4%^a^  90.9%^b^ | 99.4%^a^  85.0%^b^ | 92.5%^a^  87.0%^b^ | 99.8%^a^  89.5%^b^ | 99.2%^a^  88.1%^b^ |
| Cai SL, et al ^[11]^ | 2019 | Olympus | WLI | early ESCC | 97.8% | 85.4% | 86.4% | 97.6% | 91.4% |
| Fukuda, et al ^[12]^ | 2020 | Olympus  Fujifilm | non-ME NBI or BLI  ME NBI or BLI | ESCC | 91.1%^c^  86.7%^d^ | 5.15%^c^  89.8%^d^ | 46.1%^c^  88.6%^d^ | 92.7%^c^  88%^d^ | 63.9%^c^  88.3%^d^ |
| Horie Y, et al ^[13]^ | 2019 | Olympus | WLI NBI | ESCC, EAC | 72%^e^  86%^f^ | 79% | 39% | 95% | 99%^g^  90%^h^ |
| Luo H, et al ^[14]^ | 2019 | Olympus | WLI | upper gastrointestinal cancer | 94% | 96.1% | 88.9% | 97.9% | 95.5% |
| Guo L, et al ^[15]^ | 2019 | Olympus | NBI | early ESCC and precancerous lesions | 98.04% | 95.03% | NA | NA | NA |
| Tang D, et al ^[16]^ | 2021 | Olympus | WLI | early ESCC | 97.9% | 88.6% | 77.7% | 99.1% | 95.4% |
| Guimarães P, et al ^[17]^ | 2022 | Olympus | WLI | eosinophilic esophagitis | 87.1% | 93.6% | 83.8% | 91.1% | 91.5% |
| Tang S, et al ^[18]^ | 2022 | Olympus | WLI NBI | cancer, esophagitis and normal mucosa | 92.8% | 96.2% | 94.2% | 96.6% | 93.4% |
| Kumagai Y, et al ^[19]^ | 2022 | Olympus | Endocytoscopy | ESCC | 90.9% | 96.3% | NA | NA | 94.7% |

AI: artificial intelligence; WLI: white light imaging; DCNN: deep convolutional neural network; ESCC: esophageal squamous cell carcinoma; PPV: positive predictive value; NPV: negative predictive value; ME: magnifying endoscopy; NBI: narrow-band imaging; SESCC: superficial esophageal squamous cell carcinoma; OE: optical enhancement; DNN: deep neural network; BLI: blue-light imaging; EAC: esophageal adenocarcinoma; NA: not applicable; a: using non-ME images for early ESCC; b: using ME images for early ESCC; c: using non-ME images; d: using ME images; e: using WLI images; f: using NBI images; g: for ESCC; h: for EAC.
