# Supplementary material for: Artificial intelligence assisted detection of superficial esophageal squamous cell carcinoma in white-light endoscopic images by using a generalized system
Source: Discov Oncol. 2023 May 19;14:73. doi: 10.1007/s12672-023-00694-3 (PMC10199153; doi:10.1007/s12672-023-00694-3)
Supplement: Supplementary file 6 — Additional file 6. [file 12672_2023_694_MOESM6_ESM.docx]

Table S2. Results comparison of different backbone networks

| Network | Accuracy | Sensitivity | Specificity | AUC |
| --- | --- | --- | --- | --- |
| VGG16 | 83.5% | 83.3% | 94.7% | 0.958 |
| InceptionV3 | 83.3% | 82.1% | 94.6% | 0.955 |
| DenseNet121 | 85.4% | 83.3% | 95.1% | 0.964 |
| ResNeXt50 | 85.5% | 83.3% | **96.0 %** | 0.964 |
| ResNet50 | **85.8%** | **86.8%** | 95.8 % | **0.966** |

AUC: area under the curve
