# Supplementary material for: Artificial intelligence assisted detection of superficial esophageal squamous cell carcinoma in white-light endoscopic images by using a generalized system
Source: Discov Oncol. 2023 May 19;14:73. doi: 10.1007/s12672-023-00694-3 (PMC10199153; doi:10.1007/s12672-023-00694-3)
Supplement: Supplementary file 7 — Additional file 7. [file 12672_2023_694_MOESM7_ESM.docx]

Table S3. Results of a significant statistical test of diagnostic outcomes

|  | Internal validation set | | | | | External validation set | | | | |
| --- | --- | --- | --- | --- | --- | --- | --- | --- | --- | --- |
|  | Accuracy | Sensitivity | Specificity | PPV | NPV | Accuracy | Sensitivity | Specificity | PPV | NPV |
| AI vs. senior | 0.166 | 0.099 | 0.340 | 0.374 | 0.095 | 0.393 | <0.001 | <0.001 | 0.005 | 0.004 |
| AI vs. mid-level | <0.001 | 0.001 | <0.001 | <0.001 | <0.001 | <0.001 | 0.479 | <0.001 | <0.001 | 0.252 |
| AI vs. junior | <0.001 | 0.001 | <0.001 | <0.001 | <0.001 | <0.001 | 0.406 | <0.001 | <0.001 | 0.002 |
| senior vs. mid-level | <0.001 | 0.033 | 0.001 | 0.005 | 0.014 | <0.001 | 0.004 | <0.001 | <0.001 | <0.001 |
| senior vs. junior | <0.001 | 0.051 | <0.001 | <0.001 | 0.002 | <0.001 | 0.006 | <0.001 | <0.001 | <0.001 |
| mid-level vs. junior | <0.001 | <0.001 | <0.001 | 0.023 | 0.453 | <0.001 | 0.902 | <0.001 | <0.001 | 0.064 |

PPV: positive predictive value; NPV: negative predictive value
