# Supplementary material for: Artificial intelligence assisted detection of superficial esophageal squamous cell carcinoma in white-light endoscopic images by using a generalized system
Source: Discov Oncol. 2023 May 19;14:73. doi: 10.1007/s12672-023-00694-3 (PMC10199153; doi:10.1007/s12672-023-00694-3)
Supplement: Supplementary file 8 — Additional file 8. [file 12672_2023_694_MOESM8_ESM.docx]

Table S4. Performances of identifying different cancerous conditions in internal validation set

| Characteristics | | Groups | Accuracy | Sensitivity | Specificity | PPV | NPV |
| --- | --- | --- | --- | --- | --- | --- | --- |
| Redness | Yes | AI | 89.62% | 99.00% | 84.66% | 77.34% | 99.38% |
|  |  | Senior | 86.39% | 97.00% | 80.78% | 72.79% | 98.07% |
|  |  | Mid-level | 74.22% | 91.50% | 65.08% | 58.06% | 93.65% |
|  |  | Junior | 61.25% | 92.67% | 44.62% | 47.36% | 92.73% |
|  | No | AI | 86.45% | 100.00% | 84.66% | 46.30% | 100.00% |
|  |  | Senior | 82.24% | 93.33% | 80.78% | 39.22% | 98.92% |
|  |  | Mid-level | 67.29% | 84.00% | 65.08% | 24.13% | 96.86% |
|  |  | Junior | 48.60% | 78.67% | 44.62% | 15.76% | 94.71% |
| Nodules | Yes | AI | 86.76% | 100.00% | 84.66% | 50.85% | 100.00% |
|  |  | Senior | 83.41% | 100.00% | 80.78% | 45.34% | 100.00% |
|  |  | Mid-level | 68.73% | 91.67% | 65.08% | 29.40% | 98.02% |
|  |  | Junior | 51.44% | 94.44% | 44.62% | 21.60% | 98.53% |
|  | No | AI | 89.58% | 98.99% | 84.66% | 77.17% | 99.38% |
|  |  | Senior | 85.76% | 95.29% | 80.78% | 72.24% | 97.04% |
|  |  | Mid-level | 73.27% | 88.89% | 65.08% | 57.12% | 91.88% |
|  |  | Junior | 59.72% | 88.55% | 44.62% | 45.93% | 89.15% |
| White coating covering | Yes | AI | 87.39% | 100.00% | 84.66% | 58.57% | 100.00% |
|  |  | Senior | 83.33% | 95.12% | 80.78% | 51.87% | 98.71% |
|  |  | Mid-level | 70.43% | 95.12% | 65.08% | 37.14% | 98.40% |
|  |  | Junior | 53.62% | 95.12% | 44.62% | 27.57% | 98.03% |
|  | No | AI | 89.17% | 98.86% | 84.66% | 75.00% | 99.38% |
|  |  | Senior | 85.92% | 96.97% | 80.78% | 70.19% | 98.29% |
|  |  | Mid-level | 72.03% | 86.93% | 65.08% | 53.62% | 91.61% |
|  |  | Junior | 58.24% | 87.50% | 44.62% | 42.67% | 89.64% |
| Macroscopic types | IIb | AI | 89.58% | 98.99% | 84.66% | 77.17% | 99.38% |
|  |  | Senior | 86.11% | 96.30% | 80.78% | 72.45% | 97.66% |
|  |  | Mid-level | 73.44% | 89.40% | 65.08% | 57.25% | 92.24% |
|  |  | Junior | 59.72% | 88.55% | 44.62% | 45.93% | 89.15% |
|  | IIa/IIc | AI | 84.97% | 100.00% | 84.66% | 12.12% | 100.00% |
|  |  | Senior | 81.17% | 100.00% | 80.78% | 9.99% | 100.00% |
|  |  | Mid-level | 65.54% | 87.50% | 65.08% | 5.03% | 99.60% |
|  |  | Junior | 45.60% | 91.67% | 44.62% | 3.42% | 99.69% |
|  | Mixed type | AI | 86.32% | 100.00% | 84.66% | 44.23% | 100.00% |
|  |  | Senior | 82.39% | 95.65% | 80.78% | 37.84% | 99.35% |
|  |  | Mid-level | 67.93% | 91.31% | 65.08% | 24.13% | 98.41% |
|  |  | Junior | 50.16% | 95.65% | 44.62% | 17.67% | 99.10% |
| Locations | Upper | AI | 85.35% | 100.00% | 84.66% | 23.68% | 100.00% |
|  |  | Senior | 81.65% | 100.00% | 80.78% | 19.96% | 100.00% |
|  |  | Mid-level | 66.42% | 94.45% | 65.08% | 11.41% | 99.60% |
|  |  | Junior | 46.97% | 96.30% | 44.62% | 7.82% | 99.69% |
|  | Middle | AI | 89.26% | 100.00% | 84.66% | 73.64% | 100.00% |
|  |  | Senior | 85.43% | 96.30% | 80.78% | 68.28% | 98.07% |
|  |  | Mid-level | 72.96% | 91.36% | 65.08% | 52.82% | 94.71% |
|  |  | Junior | 58.77% | 91.77% | 44.62% | 41.96% | 92.99% |
|  | Lower | AI | 86.36% | 96.77% | 84.66% | 50.85% | 99.38% |
|  |  | Senior | 82.73% | 94.62% | 80.78% | 44.78% | 98.92% |
|  |  | Mid-level | 67.73% | 83.88% | 65.08% | 28.25% | 96.10% |
|  |  | Junior | 49.85% | 81.72% | 44.62% | 19.41% | 94.73% |
| Tumor sizes | < 2 cm | AI | 88.19% | 98.46% | 84.66% | 68.82% | 99.38% |
|  |  | Senior | 84.64% | 95.89% | 80.78% | 63.25% | 98.29% |
|  |  | Mid-level | 70.87% | 87.70% | 65.08% | 46.31% | 93.94% |
|  |  | Junior | 55.77% | 88.21% | 44.62% | 35.76% | 92.25% |
|  | ≥ 2 cm | AI | 88.49% | 100.00% | 84.66% | 68.48% | 100.00% |
|  |  | Senior | 84.79% | 96.83% | 80.78% | 62.74% | 98.71% |
|  |  | Mid-level | 71.83% | 92.07% | 65.08% | 46.74% | 96.15% |
|  |  | Junior | 56.35% | 91.54% | 44.62% | 35.86% | 94.97% |

AI: artificial intelligence; PPV: positive predictive value; NPV: negative predictive value
