# Supplementary material for: Artificial intelligence assisted detection of superficial esophageal squamous cell carcinoma in white-light endoscopic images by using a generalized system
Source: Discov Oncol. 2023 May 19;14:73. doi: 10.1007/s12672-023-00694-3 (PMC10199153; doi:10.1007/s12672-023-00694-3)
Supplement: Supplementary file 9 — Additional file 9. [file 12672_2023_694_MOESM9_ESM.docx]

Table S5. Performances of identifying different cancerous conditions in external validation set

| Characteristics | | Groups | Accuracy | Sensitivity | Specificity | PPV | NPV |
| --- | --- | --- | --- | --- | --- | --- | --- |
| Redness | Yes | AI | 90.85% | 92.99% | 88.89% | 88.46% | 93.26% |
|  |  | Senior | 88.44% | 97.93% | 79.75% | 81.59% | 97.68% |
|  |  | Mid-level | 75.32% | 94.48% | 57.78% | 67.24% | 92.50% |
|  |  | Junior | 63.15% | 93.98% | 34.90% | 57.02% | 87.71% |
|  | No | AI | 88.46% | 86.96% | 88.89% | 68.97% | 96.00% |
|  |  | Senior | 82.82% | 93.62% | 79.75% | 56.78% | 97.78% |
|  |  | Mid-level | 64.23% | 86.96% | 57.78% | 36.84% | 94.28% |
|  |  | Junior | 47.18% | 90.44% | 34.90% | 28.35% | 93.28% |
| Nodules | Yes | AI | 90.22% | 96.51% | 88.89% | 64.84% | 99.17% |
|  |  | Senior | 83.30% | 100.00% | 79.75% | 51.21% | 100.00% |
|  |  | Mid-level | 64.97% | 98.84% | 57.78% | 33.26% | 99.60% |
|  |  | Junior | 46.03% | 98.45% | 34.90% | 24.42% | 99.17% |
|  | No | AI | 89.63% | 90.37% | 88.89% | 89.05% | 90.23% |
|  |  | Senior | 87.90% | 96.05% | 79.75% | 82.59% | 95.29% |
|  |  | Mid-level | 74.57% | 91.36% | 57.78% | 68.39% | 88.02% |
|  |  | Junior | 63.38% | 91.85% | 34.90% | 58.59% | 82.76% |
| White coating covering | Yes | AI | 90.24% | 96.55% | 88.89% | 65.12% | 99.17% |
|  |  | Senior | 83.26% | 99.62% | 79.75% | 51.40% | 99.90% |
|  |  | Mid-level | 64.94% | 98.28% | 57.78% | 33.39% | 99.40% |
|  |  | Junior | 46.00% | 97.70% | 34.90% | 24.50% | 98.54% |
|  | No | AI | 89.67% | 90.44% | 88.89% | 89.13% | 90.23% |
|  |  | Senior | 87.99% | 96.16% | 79.75% | 82.71% | 95.38% |
|  |  | Mid-level | 74.72% | 91.55% | 57.78% | 68.60% | 88.15% |
|  |  | Junior | 63.59% | 92.08% | 34.90% | 58.83% | 83.31% |
| Macroscopic types | IIb | AI | 89.64% | 90.46% | 88.89% | 88.06% | 91.14% |
|  |  | Senior | 87.44% | 95.92% | 79.75% | 81.11% | 95.57% |
|  |  | Mid-level | 73.64% | 91.15% | 57.78% | 66.17% | 88.69% |
|  |  | Junior | 61.79% | 91.46% | 34.90% | 56.08% | 83.54% |
|  | IIa/IIc | AI | 88.73% | 85.71% | 88.89% | 28.57% | 99.17% |
|  |  | Senior | 80.52% | 95.24% | 79.75% | 19.63% | 99.69% |
|  |  | Mid-level | 59.51% | 92.86% | 57.78% | 10.23% | 99.40% |
|  |  | Junior | 37.87% | 95.24% | 34.90% | 7.08% | 99.38% |
|  | Mixed type | AI | 90.26% | 95.92% | 88.89% | 67.63% | 98.90% |
|  |  | Senior | 83.70% | 100.00% | 79.75% | 54.46% | 100.00% |
|  |  | Mid-level | 65.61% | 97.96% | 57.78% | 36.01% | 99.20% |
|  |  | Junior | 47.19% | 97.96% | 34.90% | 26.81% | 98.66% |
| Locations | Upper | AI | 89.22% | 93.55% | 88.89% | 39.19% | 99.45% |
|  |  | Senior | 80.96% | 96.77% | 79.75% | 26.81% | 99.69% |
|  |  | Mid-level | 60.21% | 91.94% | 57.78% | 14.27% | 99.00% |
|  |  | Junior | 38.84% | 90.32% | 34.90% | 9.61% | 98.19% |
|  | Middle | AI | 89.76% | 90.76% | 88.89% | 87.80% | 91.60% |
|  |  | Senior | 87.75% | 96.83% | 79.75% | 80.83% | 96.62% |
|  |  | Mid-level | 74.22% | 92.86% | 57.78% | 65.98% | 90.93% |
|  |  | Junior | 62.34% | 93.47% | 34.90% | 55.95% | 86.99% |
|  | Lower | AI | 89.67% | 93.67% | 88.89% | 62.18% | 98.63% |
|  |  | Senior | 82.51% | 96.63% | 79.75% | 48.23% | 99.18% |
|  |  | Mid-level | 63.43% | 92.41% | 57.78% | 29.96% | 97.54% |
|  |  | Junior | 44.42% | 93.25% | 34.90% | 21.91% | 96.64% |
| Tumor sizes | < 2 cm | AI | 89.57% | 90.69% | 88.89% | 83.27% | 93.99% |
|  |  | Senior | 85.63% | 95.28% | 79.75% | 74.17% | 96.52% |
|  |  | Mid-level | 69.79% | 89.48% | 57.78% | 56.34% | 90.72% |
|  |  | Junior | 55.88% | 90.28% | 34.90% | 45.88% | 86.82% |
|  | ≥ 2 cm | AI | 90.25% | 92.64% | 88.89% | 82.63% | 95.49% |
|  |  | Senior | 86.58% | 98.56% | 79.75% | 73.53% | 98.98% |
|  |  | Mid-level | 72.48% | 96.11% | 57.78% | 56.53% | 96.45% |
|  |  | Junior | 57.18% | 96.25% | 34.90% | 45.85% | 94.75% |

AI: artificial intelligence; PPV: positive predictive value; NPV: negative predictive value
